# Retraction: A meta-analysis and of clinical values of 11 blood biomarkers, such as AFP, DCP, and GP73 for diagnosis of hepatocellular carcinoma

**DOI:** 10.1080/07853890.2024.2415160

**Published:** 2024-10-16

We, the Editors and Publisher of the journal *Annals of Medicine*, have retracted the following article:

Bing-yao Pang, Yan Leng, Xiaoli Wang, Yi-qiang Wang & Li-hong Jiang (2023) A meta-analysis and of clinical values of 11 blood biomarkers, such as AFP, DCP, and GP73 for diagnosis of hepatocellular carcinoma, *Annals of Medicine*, 55:1, 42-61, DOI: http://doi.org/10.1080/07853890.2022.2153163

Since publication, concerns have been raised about the integrity of the data presented in the article. When approached for an explanation, the authors have been unable to provide sufficient details to address the concerns raised. In addition, it has come to our attention that the authorship list for this manuscript was significantly changed after the submission of the article. We have contacted the authors for a full explanation but have not received a satisfactory explanation.

As determining authorship and verifying the validity of published work is core to the integrity of the scholarly record, we are therefore retracting the article. The corresponding author listed in this publication has been informed.

We have been informed in our decision-making by our editorial policies and the COPE guidelines. 

The retracted article will remain online to maintain the scholarly record, but it will be digitally watermarked on each page as ‘Retracted’.

